# The Level and Significance of Circulating Angiotensin-III in Patients with Coronary Atherosclerosis

**DOI:** 10.1155/2021/1704762

**Published:** 2021-09-21

**Authors:** Guoqing Yao, Wenjing Li, Wenzhao Liu, Jingbo Xing, Cheng Zhang

**Affiliations:** ^1^The Key Laboratory of Cardiovascular Remodeling and Function Research, Chinese Ministry of Education and Chinese Ministry of Public Health, Qilu Hospital, Cheeloo College of Medicine, Shandong University, Jinan, Shandong 250012, China; ^2^Fine Arts School of Shandong University, Jinan, Shandong 250012, China

## Abstract

**Objective:**

Angiotensin-III (Ang-III) is the downstream product of angiotensin-II (Ang-II) metabolized by aminopeptidase A (APA). At present, the research of Ang-III mainly concentrates on hypertension and the central renin-angiotensin system (RAS). However, few studies have focused on the relationship between Ang-III and coronary atherosclerosis (CAS).

**Methods and Results:**

Plasma Ang-III and APA levels were measured by the enzyme-linked immunosorbent assay (ELISA) in 44 normal subjects and 84 patients confirmed as having CAS by coronary angiography. Circulating Ang-III levels were significantly lower in patients with CAS than in normal controls (*P* = 0.013). APA levels were slightly lower in the CAS group (*P* = 0.324). According to the severity of atherosclerosis, CAS patients were divided into two groups. Compared with the controls, the APA and Ang-III levels were lower in the high scoring group and APA decreased significantly.

**Conclusions:**

Circulating Ang-III levels were reduced in patients with CAS, and the possible reason may be related to the decrease in the APA level.

## 1. Introduction

As an important component of the renin-angiotensin system (RAS), angiotensin-II (Ang-II) has been considered to play a crucial role in the occurrence and development of coronary atherosclerosis (CAS) [1, 2]. This is based on the strong position of the classical axis of angiotensinogen/renin/angiotensin-converting enzyme (ACE)/Ang-II/Ang-II type 1 receptor (AT1R)/aldosterone in RAS. Derived from the metabolism of this classic axis, there are other axes including the Ang-I/Ang-II/aminopeptidase A (APA)/Ang-III/AT2R/NO/cGMP axis and the Ang-I/Ang-II/ACE2/Ang-(1-7)/Mas receptor axis [3]. Hence, the peptides or enzymes in the these axes, such as Ang-III, Ang-(1-7), and ACE2, have aroused widespread concern [2, 4–6]. Ang-III is normally produced by the intrarenal metabolism of Ang-II by APA, which can be further processed into Ang-(3-8), namely, Ang-IV. After the biological activity of Ang-III was discovered, many studies thought that it had similar function with Ang-II [7]. Since Ang-III has a high clearance rate in peripheral circulation and its effects would be masked by Ang-II under normal physiological conditions [7, 8], little is known about the level and influence of Ang-III in the peripheral circulation of patients with CAS. This study was aimed at exploring the plasma level of Ang-III in patients with CAS. It is expected to further improve the research of RAS in coronary artery disease and provide new ideas for the treatment and prevention of coronary atherosclerosis.

## 2. Materials and Methods

### 2.1. Participants

A total of 84 patients with CAS confirmed by coronary angiography during hospitalization in the Department of Cardiology, Qilu Hospital of Shandong University, were prospectively collected. During the same period, 44 normal people were collected from the surgery department of our hospital as controls. The CAS group was required to meet the following conditions: (1) patients who needed coronary angiography due to new-onset angina attack or have a history of angina attack and (2) those that had atherosclerosis confirmed by coronary angiography. Patients with any of the following were excluded: (1) having history of hypertension or newly discovered hypertension after admission (the systolic blood pressure ≥ 140 mmHg or diastolic blood pressure ≥ 90 mmHg), (2) taking angiotensin-converting enzyme inhibitors (ACEIs) or angiotensin receptor blockers (ARBs) within 1 month, (3) having moderate or severe anemia (hemoglobin < 90 g/l), and (4) having severe rheumatic, infectious, hematologic, or other systemic diseases. The controls from the surgery department met the following conditions: (1) all included subjects only suffered from diseases related to bone and joint degeneration, such as lumbar disc herniation. Subjects with any chronic disease were excluded. (2) Patients are not taking medicine within 1 month. This study was approved by the ethics committee of Qilu Hospital of Shandong University, and all patients provided written informed consent before participating.

### 2.2. Anthropometric Data and Blood Sampling

All samples of the two groups were collected prior to surgery or medication. All enrolled patients had a fasting blood sample drawn from the antecubital vein after fasting for 8 hours, and these samples were sent to the department of hospital laboratory for the detection of cardiac enzyme, blood glucose, lipid profile, serum creatinine, urea nitrogen, and other indicators. In addition, 5 ml of fresh blood was collected using an anticoagulant tube containing EDTA and centrifuged at 3000 rpm for 15 minutes at 4°C within one hour after collection. Plasma was collected in 1.5 ml EP tubes and stored at -80°C until analysis. Plasma Ang-III levels were determined using commercial enzyme-linked immunosorbent assay (ELISA) kits (BioVision, San Francisco, USA). Plasma APA levels were tested using ELISA kits from Abbexa (Cambridge, UK). All subjects received electrocardiogram and echocardiography examination. Anthropometric data such as gender, age, heart rate, smoking history, and blood pressure were obtained retrospectively from the hospital medical record query system.

### 2.3. Coronary Angiography

Coronary angiography is to insert a catheter through the femoral or radial artery of the thigh to the ascending aorta and then explore the left or right coronary ostia. Inject an iodine contrast agent to visualize the coronary arteries, which can clearly reveal the coronary artery anatomic deformity and the location, degree, or scope of the obstructive lesions. The coronary angiography results were evaluated by two specialists independently. Depending on the location, severity of the stenosis, and clinical symptoms, specialists may perform balloon dilatation or stenting depending on the situation through this procedure.

### 2.4. Statistical Analysis

All statistical analyses were performed by using SPSS 25.0. Data conforming to normal distribution were shown as mean values ± standard deviation ($\bar{x}\pm S$). Data that did not conform to the normal distribution were log-transformed. Differences among continuous variables between two groups were evaluated using two-sample *t*-tests. One-way analysis of variance (ANOVA) was used to compare the differences among the three groups. Categorical variables were expressed as frequencies (%) and tested by the chi-square test. A *P* value < 0.05 was considered statistically significant.

## 3. Results

### 3.1. Clinical and Biochemical Characteristics of the Study Population at Baseline

The demographic and biochemical characteristics of the subjects are summarized in Table 1. A total of 128 subjects, including 84 patients with CAS and 44 control subjects, were included in the study. There were no differences in gender, smoking history, heart rate, and age. For biochemical characteristics, there were no differences in serum potassium, Scr, BUN, blood glucose, and TG. The LDL-c, TC, and HDL-c were lower in patients with CAS compared with control subjects. For cardiac enzyme, the cTnI was higher in the CAS group than in controls. There was no difference in blood pressure between the two groups. All CAS subjects received coronary angiography. Forty-five patients received coronary stent implantation, six patients underwent drug coated balloon dilatation, and three patients needed coronary artery bypass grafting. The remaining 30 patients did not receive treatment during coronary angiography.

### 3.2. Plasma Levels of Ang-III and APA

Ang-III levels were significantly lower in patients with coronary atherosclerosis (5.6 ± 1.9 ng/ml) than in controls (6.5 ± 2.1 ng/ml) (*P* = 0.013) (Table 2). The APA levels were slightly lower in the CAS group (*P* = 0.324).

### 3.3. Correlation between Ang-III and the Severity of Atherosclerosis

To explore whether there is a relationship between the Ang-III level and severity of CAS, we used the Gensini score [9] to quantify the severity of CAS. Higher scores mean more severe CAS. We sorted the calculated scores and divided them into two groups in order (Table 3). The APA (1.38 ± 0.56 ng/ml) and Ang-III (4.25 ± 1.41 ng/ml) levels were lower in the high scoring group, and APA decreased significantly (*P* = 0.037). Ang-III levels displayed the same trend though it was not significant.

## 4. Discussion

Ang-III has been widely concerned since it was found to be a vasoactive peptide. Because it is generated by removing N-terminal single amino acid from Ang-II by aminopeptidase, the initial researchers first considered that its function is similar to that of Ang-II. Starting from this study point, researchers have successively found its role in sodium and water regulation, promotion of aldosterone release, blood pressure regulation, proinflammation, etc. [7, 10–14]. And most of its effects are thought to be mediated by AT1R. In central RAS, Ang-III is considered to be an essential substance acting on AT1R to exert a pressor effect, and its effect even exceeds that of Ang-II [6]. However, with the discovery of the Ang-II/APA/Ang-III/AT2R/NO/cGMP axis and the study of the role of AT2R activation in cardiovascular disease [3, 15, 16], it was found that Ang-III has positive effects independent of Ang-II. Studies have found that AT2R activation can produce natriuresis, vasodilation, anti-inflammation, and other antagonistic effects of AT1R, and accumulating evidence indicates that Ang-III is an important endogenous AT2R agonist in the kidney and coronary artery [17, 18].

It is generally believed that circulating Ang-III has a short half-life, and its actions are masked by Ang-II in a normal physiological state due to the absolute superiority of AT1R distribution, so its function has not been paid much attention to. To explore whether there is a relationship between endogenous circulating Ang-III and CAS, we examined the levels of Ang-III in the peripheral circulation. The results showed that the levels of Ang-III in the CAS group were significantly lower and the APA levels were slightly lower than those in controls. To further explore whether Ang-III level is associated with the severity of CAS, we used the Gensini score to quantify the severity of CAS. We can see that APA levels were significantly lower in the high scoring group, and Ang-III levels displayed the same trend though it was not significant. Regarding this result, we have several hypotheses as follows. (1) In pathological conditions such as myocardial infarction [19], it has been demonstrated that AT2R expression is upregulated. In a myocardial ischemia-reperfusion injury model [20], Ang-III combines with AT2R to produce NO and cGMP, which are involved in the early protection of myocardial ischemia. Ang-III, as an important AT2R agonist, may be more likely to bind to and act in the ischemic environment caused by coronary atherosclerosis. Secondly, Ang-III can be metabolized into Ang-IV by aminopeptidase N, and Ang-IV has also been studied as having a myocardial protective effect [21]. The consumption of Ang-III can be increased either by combining with AT2R directly or by metabolizing to Ang-IV. We also speculate that Ang-III may increase in the early stage of atherosclerosis in a compensatory manner. Since the patients in our study were mostly in the late stage, this point could be explored in future study. (2) We have known that Ang-III is mainly derived from the metabolism of Ang-II by APA and will be metabolized into Ang-IV by APN. In the peripheral circulation of patients with essential hypertension [22], APA activity is attenuated and APN activity is enhanced, presumably leading to decreased Ang-III production and increased metabolism. We get inspiration from this research, so we measured the concentration of APA in both groups. The results showed that the APA levels in the CAS group were slightly lower than those in the control group. Then, we divided the CAS group into two groups according to their severity. The APA levels decreased significantly in the severe group. Therefore, we speculated that the decrease in Ang-III might be related to the decrease in APA. And this shows a correlation between Ang-III and the severity of CAS. (3) In addition to the concentration changes due to the upstream and downstream factors speculated above, Ang-III may itself decrease expression under coronary atherosclerotic conditions.

Of course, there are also limitations in our study. Firstly, due to funding constraints, we choose ELISA kits to detect the samples. The overall levels of Ang-III were higher than theoretical ones perhaps due to its high sensitivity. It is better to choose liquid chromatography-tandem mass spectrometry (LC-MS/MS) for accurate quantification [23]. Secondly, in this experiment, we did not focus much attention on Ang-II. Ang-II is considered to have proatherogenic effects, and the plasma Ang-II levels have been confirmed to be higher in patients with coronary atherosclerosis [2, 24]. So we did not assume that the decrease in Ang-III was due to the decrease in Ang-II. But if we measured the concentration of rennin, angiotensinogen, and Ang-II, the results would be more convincing. Thirdly, the sample size was relatively small and the enrolled patients were mostly in the late stage of atherosclerosis, which leads to limitations of more analysis. We aim to recruit more subjects and enlarge the sample size.

In conclusion, our present study found that the Ang-III levels were decreased in patients with CAS for the first time, which might be related to the decrease in APA. This requires a larger sample size and more precise testing methods for confirmation.

## Figures and Tables

**Table 1:** The demographic and biochemical characteristics of the study population.

	Controls (*n* = 44)	CAS (*n* = 84)	*P* value
Gender (female/male)	18/26	31/53	0.658
Age (years)	54 ± 12	57 ± 8	0.641
Smoking (%)	34	45	0.995
Heart rate	75 ± 6	73 ± 11	0.191
TC (mmol/l)	4.6 ± 1.2	3.7 ± 1.1	<0.001
LDL-c (mmol/l)	2.6 ± 0.8	2.0 ± 0.9	<0.001
TG (mmol/l)	1.4 ± 0.6	1.6 ± 0.9	0.33
BUN (*μ*mol/l)	5.6 ± 1.5	5.0 ± 1.9	0.054
Scr (mmol/l)	70 ± 14	74 ± 14	0.087
HDL-c (mmol/l)	1.2 ± 0.3	1.0 ± 0.2	0.002
SBP	122.9 ± 8.6	122.0 ± 9.6	0.601
DBP	74.5 ± 7.4	72.0 ± 8.0	0.104
Blood glucose	5.2 ± 0.7	5.5 ± 1.5	0.268
cTnI	0 ± 15.49	0.78 ± 93.32	<0.001
CK-MB	1.33 ± 1.0	1.7 ± 2.4	0.316
CK	80.1 ± 48.6	72.8 ± 54.2	0.612
Serum potassium	4.2 ± 0.3	4.1 ± 0.4	0.427

CAS: coronary atherosclerosis; TC: total cholesterol; LDL-c: low-density lipoprotein cholesterol; TG: triglyceride; Scr: serum creatinine; BUN: blood urea nitrogen; HDL-c: high-density lipoprotein cholesterol; SBP: systolic blood pressure; DBP: diastolic blood pressure; cTnI: cardiac troponin I; CK: creatine kinase; CK-MB: creatine kinase isoenzyme.

**Table 2:** The plasma levels of Ang-III and APA in CAS patients and controls.

	Controls (*n* = 44)	CAS (*n* = 84)	*P* value
Ang-III (ng/ml)	6.5 ± 2.1	5.6 ± 1.9	0.013
APA (ng/ml)	1.92 ± 1.15	1.74 ± 0.90	0.324

**Table 3:** The plasma levels of Ang-III and APA in different scoring groups.

	Low scoring (*n* = 42)	High scoring (*n* = 42)	Controls (*n* = 44)	*P* value
Ang-III (ng/ml)	5.16 ± 2.07	4.25 ± 1.41	6.5 ± 2.1	0.11
APA (ng/ml)	1.98 ± 1.12	1.38 ± 0.56	1.92 ± 1.15	0.037

## Data Availability

All data generated or analysed during this study are included in this published article. The data have been deposited in Figshare: (DOI:10.6084/m9.figshare.15657402). Requests for material should be made to the corresponding authors.
